# ExPeCT: a randomised trial examining the impact of exercise on quality of life in men with metastatic prostate cancer

**DOI:** 10.1007/s00520-023-07740-4

**Published:** 2023-04-22

**Authors:** Gráinne Sheill, Lauren Brady, Brian Hayes, Anne-Marie Baird, Emer Guinan, Rishabh Vishwakarma, Caroline Brophy, Tatjana Vlajnic, Orla Casey, Verena Murphy, John Greene, Emma Allott, Juliette Hussey, Fidelma Cahill, Mieke Van Hemelrijck, Nicola Peat, Lorelei Mucci, Moya Cunningham, Liam Grogan, Thomas Lynch, Rustom P. Manecksha, John McCaffrey, Dearbhaile O’Donnell, Orla Sheils, John O’Leary, Sarah Rudman, Ray McDermott, Stephen Finn

**Affiliations:** 1 Discipline of Physiotherapy, School of Medicine, Trinity College Dublin, Trinity Centre for Health Sciences, St James’s Hospital, Dublin 8, Ireland; 2 Department of Histopathology and Morbid Anatomy, Trinity Translational Medicine Institute, Trinity College Dublin, Dublin, Ireland; 3 Department of Histopathology, Cork University Hospital, Cork, Ireland; 4 Department of Pathology, University College Cork, Cork, Ireland; 5 School of Medicine, Trinity College Dublin, Dublin, Ireland; 6 School of Computer Science and Statistics, Trinity College Dublin, Dublin 2, Ireland; 7 Institute of Pathology, University Hospital Basel, Basel, Switzerland; 8 Cancer Trials Ireland, Dublin, Ireland; 9 Centre for Cancer Research and Cell Biology, Queen’s University Belfast, Northern Ireland, Belfast, UK; 10 School of Cancer and Pharmaceutical Sciences, Translational Oncology and Urology Research (TOUR), King’s College London, London, UK; 11 Guy’s and St Thomas’ NHS Foundation Trust, London, UK; 12 Harvard T.H. Chan School of Public Health, Boston, MA USA; 13 Department of Radiation Oncology, St Luke’s Hospital, Dublin, Ireland; 14 Department of Oncology, Beaumont Hospital, Dublin, Ireland; 15 Department of Urology, St James’s Hospital, Dublin, Ireland; 16 Department of Surgery, Trinity College Dublin, Dublin, Ireland; 17 Department of Oncology, Mater Misericordiae Hospital, Dublin, Ireland; 18 HOPE Directorate, St James’s Hospital, Dublin, Ireland; 19 Department of Histopathology, St James’s Hospital, Dublin, Ireland; 20 Department of Oncology, Tallaght University Hospital, Dublin, Ireland

**Keywords:** Exercise, Physical activity, Quality of life, Advanced cancer

## Abstract

**Purpose:**

All patients living with cancer, including those with metastatic cancer, are encouraged to be physically active. This paper examines the secondary endpoints of an aerobic exercise intervention for men with metastatic prostate cancer.

**Methods:**

ExPeCT (Exercise, Prostate Cancer and Circulating Tumour Cells), was a multi-centre randomised control trial with a 6-month aerobic exercise intervention arm or a standard care control arm. Exercise adherence data was collected via heart rate monitors. Quality of life (FACT-P) and physical activity (self-administered questionnaire) assessments were completed at baseline, at 3 months and at 6 months.

**Results:**

A total of 61 patients were included (69.4 ± 7.3 yr, body mass index 29.2 ± 5.8 kg/m^2^). The median time since diagnosis was 34 months (IQR 7–54). A total of 35 (55%) of participants had > 1 region affected by metastatic disease. No adverse events were reported by participants. There was no effect of exercise on quality of life (Cohen’s *d* =  − 0.082). Overall adherence to the supervised sessions was 83% (329 out of 396 possible sessions attended by participants). Overall adherence to the non-supervised home exercise sessions was 72% (months 1–3) and 67% (months 3–6). Modelling results for overall physical activity scores showed no significant main effect for the group (*p*-value = 0.25) or for time (*p*-value = 0.24).

**Conclusion:**

In a group of patients with a high burden of metastatic prostate cancer, a 6-month aerobic exercise intervention did not lead to change in quality of life. Further exercise studies examining the role of exercise for people living with metastatic prostate cancer are needed.

**Trial Registration:**

The trial was registered at clinicaltrials.gov (NCT02453139) on May 25th 2015.

**Supplementary information:**

The online version contains supplementary material available at 10.1007/s00520-023-07740-4.

## Introduction

Approximately 10–20% of men with prostate cancer present with metastatic disease, and as many as 80% will develop bone metastases due to disease progression [1, 2]. Patients are now living longer with metastatic cancer, and the need for physical rehabilitation is increasing, to help counteract the adverse effects of long-term systemic treatments on strength, fatigue, and physical functioning [3]. Additionally, exercise is emerging as a synergistic medicine (i.e. increasing the potency or effectiveness of concomitantly applied therapies) and targeted medicine (i.e. exerting its own systemic and localised anticancer effects) to underpin delays in disease progression and improvements in survival for patients with advanced cancer [4, 5]. Therefore, it is essential to devise and implement exercise interventions suitable for all patients with advanced cancer, including those previously excluded from participation such as patients with bone metastases.

Exercise interventions for patients with bone metastases are associated with positive physical and self-reported outcomes [4]. International exercise oncology guidelines now suggest that all patients living with cancer, including those with bone metastases, should avoid inactivity and achieve 150 min of weekly moderate intensity exercise when possible [6–8]. Therefore, there is a need to investigate how patients with metastatic disease tolerate physical activity programmes, and explore the benefits associated with such exercise programmes. The ExPeCT (Exercise, Prostate Cancer and Circulating Tumour Cells) trial was conceived to elucidate the relationship between exercise, platelet cloaking (the “cloaking” of tumour cells by adherent platelets), and circulating tumour cells in patients with metastatic prostate cancer [9]. A full report detailing the results regarding circulating tumour cells (the primary outcome) can be viewed elsewhere [10]. The purpose of this manuscript is to report on the secondary outcomes of the ExPeCT trial, regarding the effect of a 6-month aerobic exercise intervention, prescribed in line with guidelines for aerobic activity in cancer survivors, on quality of life in men diagnosed with metastatic prostate cancer [6]. Additionally, the safety of a structured aerobic exercise intervention and effects on physical activity levels of participants will be explored.

## Methods

### Study design

The ExPeCT trial was an international multi-centre two-armed randomised controlled trial (RCT). Men living with metastatic prostate cancer were randomly assigned to either a 6-month aerobic exercise programme or to the control arm. Patients were recruited between October 2014 and until study completion in March 2017.

The study protocol has been described previously (9). In summary, eligibility criteria included the folowing: (1) patients aged ≥ 18 years and male, (2) histologically confirmed diagnosis of prostate adenocarcinoma, (3) M1 metastatic disease as confirmed by computed tomography (CT)/magnetic resonance imaging (MRI) or by bone scan, excluding patients who only had nodal metastatic disease, (4) stable medical condition, including the absence of acute exacerbations of chronic illnesses, serious infections, or major surgery within 28 days prior to randomisation, (5) capable of participating safely in the proposed exercise as assessed and signed off by a treating physician involved in ExPeCT recruitment. Exclusion criteria included the following: (1) patients with a history of radical prostatectomy, (2) patients with other known malignancy (except non-melanoma skin cancers or fully excised carcinoma in situ at any site).

Participants were enrolled by appropriate staff at the medical oncology clinics at each of the six recruiting sites in Dublin, Ireland, and London, UK. Written informed consent was obtained by clinic staff or a member of the ExPeCT research team according to the requirements of the International Conference on Harmonisation—Good Clinical Practice. Randomisation was based on a computer-generated algorithm held and controlled by an independent gatekeeper to conceal allocation. Sample size was calculated based on the primary outcome of circulating tumour cells [9].

### Ethical approval

The ExPeCT study was approved by ethical review committees at each of the six recruiting sites in Ireland and in the UK. The trial is registered at clinicaltrials.gov (NCT02453139).

### Measures

Assessments were completed at baseline (T0), at 3 months (T3) and at 6 months (T6). Demographic details were collected using a standardised questionnaire derived from the Harvard Health Professionals Follow-up study [5]. Quality of life was measured using the Functional Assessment of Cancer Therapy – Prostate (FACT-P) questionnaire. A low FACT-P score reflects a lower health-related QOL and more concerns specific to prostate cancer and its treatment [11]. Sleep was measured by the Pittsburgh Sleep Quality Index [12]. Stress was measured with the Perceived Stress Scale – 4 [13]. Depression was measured with the PHQ-9. A self-administered physical activity questionnaire derived from the Harvard Health Professional’s Follow-up study was used to measure physical activity levels. Full details on all measures have been described previously [9]. Exercise adherence data was collected via Polar heart rate monitors, worn by the patient for every exercise session undertaken, and participant completed physical activity diaries to record daily physical activity levels. Adherence (tolerability) outcomes were as follows: rates of lost-to-follow-up (LTF), number completing follow-up assessments; attendance, adherence (percentage of total sessions attended to planned sessions); permanent treatment discontinuation, permanent discontinuation of aerobic training before week 24; early session termination, at least one session requiring early termination.

### Intervention

#### Exercise programme

The ExPeCT exercise programme has been described previously [8]. To summarise, the exercise group participated in a 6-month moderate to vigorous intensity aerobic exercise programme comprising a weekly class and a home-based aerobic exercise programme. Patients could self-select the exercise modality used, e.g. treadmills, stationary bikes. Participants exercised to a prescribed heart rate range which progressed in intensity and duration during the intervention, based on self-reported baseline activity levels [14].

Exercise intensity was prescribed using individualised heart rate reserve (HRR) ranges in accordance with the American College of Sports Medicine (ACSM) guidelines [14]. Patients were also encouraged to use the Borg Breathlessness Scale to gauge exercise intensity. Exercise was prescribed to avoid loading bones at areas of the body with metastatic lesions.

The occurrence and severity of any incidents were recorded by the chartered physiotherapist from the time of consent to completion of the programme at 6 months on a standardised reporting form.

From T0 to T3, patients attended weekly exercise classes. Polar heart rate data was downloaded at each weekly class. From T3 to T6, patients conducted unsupervised home-based exercise only, and attended research centres once monthly to download Polar heart rate data. The control group was given the standard physical activity recommendations for cancer survivors and continued to receive usual standard of care. Control participants were offered an exercise advice session following completion of the T6 assessment. Further detail of the exercise intervention is given in the published protocol [9].

### Statistical analysis

Parts of the statistical analyses were conducted using the IBM Statistical Package for the Social Sciences (SPSS) (Version 20) for Windows (IBM, Somers, NY, USA). An intention-to-treat (ITT) approach was used. Descriptive statistics were used to profile the demographic data and disease characteristics as well as quality of life, depression, and stress scores. Baseline values for demographic data, disease characteristics, and quality of life outcome measures between the exercise and control groups were compared using either a *t*-test or a *χ*^2^-test.

Statistical analyses related to modelling the treatment effect were conducted using the programming language R [15]. The responses considered for the analysis were quality of life (QOL), depression, stress, sleep score, BMI, systolic and diastolic blood pressure, and physical activity. An initial analysis was carried out to find the proportion of patients with missing values for each of these responses and as the proportion of missing values was found to be around 20%, they were imputed using a version of the closest match method described in Elliot and Hawthorne [16].

Linear mixed models assuming Gaussian errors were used to model each response as a function of the main effects: group (a factor with two levels — standard care control and aerobic exercise intervention) and time (a factor with three levels — baseline, 3 months and 6 months). An interaction term (group × time) was also tested for each response. The error term in each model allowed for the correlated nature of the repeated measures recorded on each patient. Different covariance structures were tested and the best structure for each response was selected using restricted maximum likelihood estimation and the corrected Akaike information criterion (AICc) [17]. After deciding on a covariance structure for the response, the main effects and the interaction effect were estimated using maximum likelihood and their significance was tested using *F*-tests. Cohen’s effect sizes [18] were also estimated to find the size of the effect of exercise on the responses.

The effect of exercise adherence on the response was also investigated with adherence to the exercise programme being calculated as the ratio of total completed sessions to total prescribed sessions, expressed as a percentage. The association between exercise adherence and each individual response was tested separately using Pearson’s product-moment correlation. All the tests involved a two-sided significance level of *a* = 0.05.

## Results

**Fig. 1:**
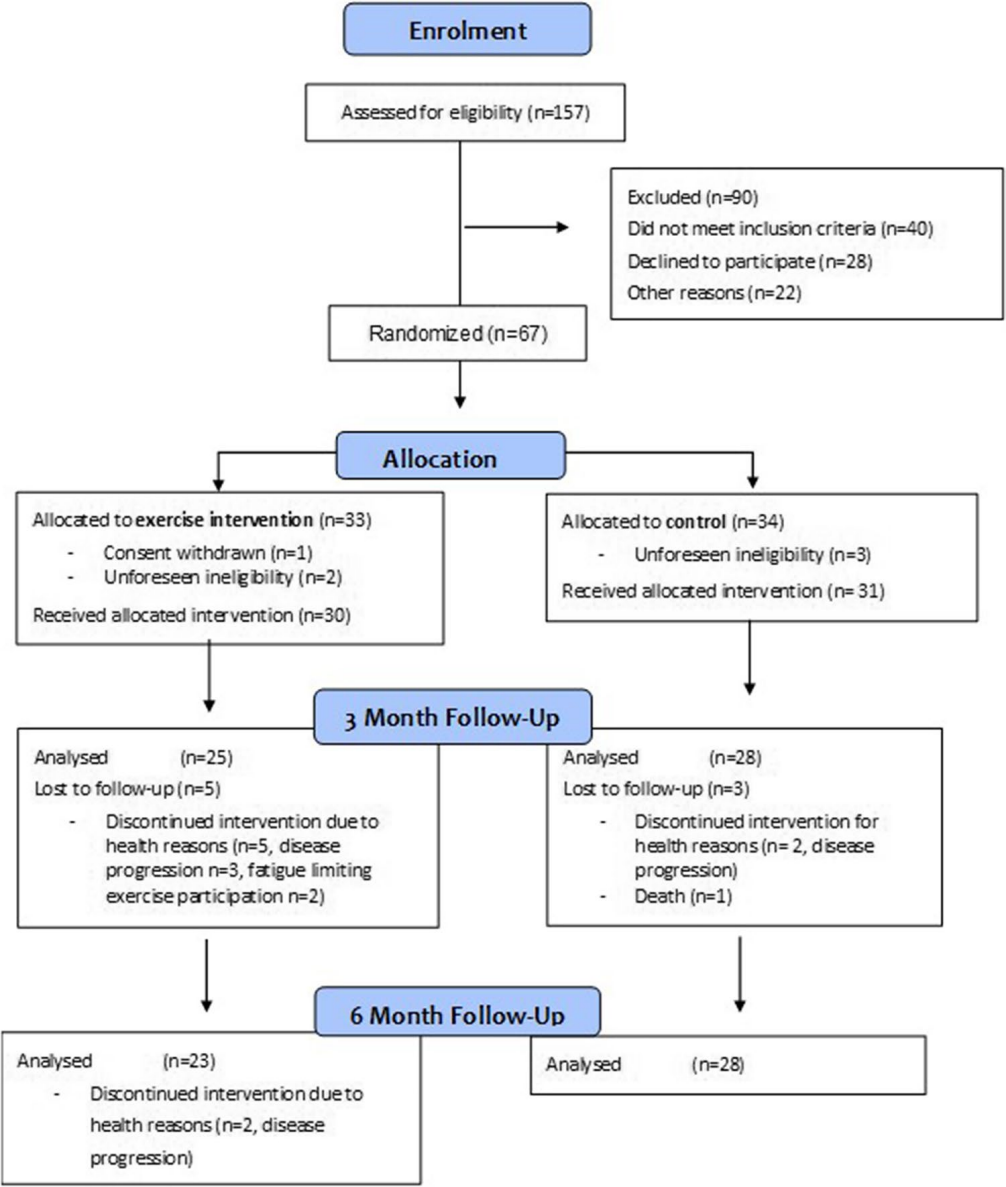
CONSORT Diagram

Between October 2014 and March 2017, 157 patients were screened for participation in ExPeCT, of which 67 patients were consented and randomised to the trial, representing a recruitment rate of 43% (Fig. 1). A total of 33 participants were randomly assigned to the exercise group and 34 participants were randomly assigned to the control group. A total of 53 (86%) of participants completed the 3-month assessment and 51 (84%) of the participants completed the 6-month assessment. The proportion of patients lost to follow-up was higher in the exercise group (24%) than in the control group (14%) (*p* = 0.048). Reasons for loss to follow-up are detailed in Fig. 1.

Patient demographic characteristics are presented in Table 1. Groups were comparable at baseline for demographic characteristics with the exception of the number of smokers, which was significantly higher in the exercise group.

Patients’ clinical characteristics are presented in Table 2. At baseline, physical activity levels were comparable in both groups. Patients had extensive metastatic bone disease characterised by > 1 regions affected by metastatic lesions (Table 2). At baseline groups were comparable at baseline for disease characteristics with the exception of a number of patients actively receiving radiation therapy, which were significantly higher in the exercise group.

### Intervention adherence (tolerability)

**Fig. 2:**
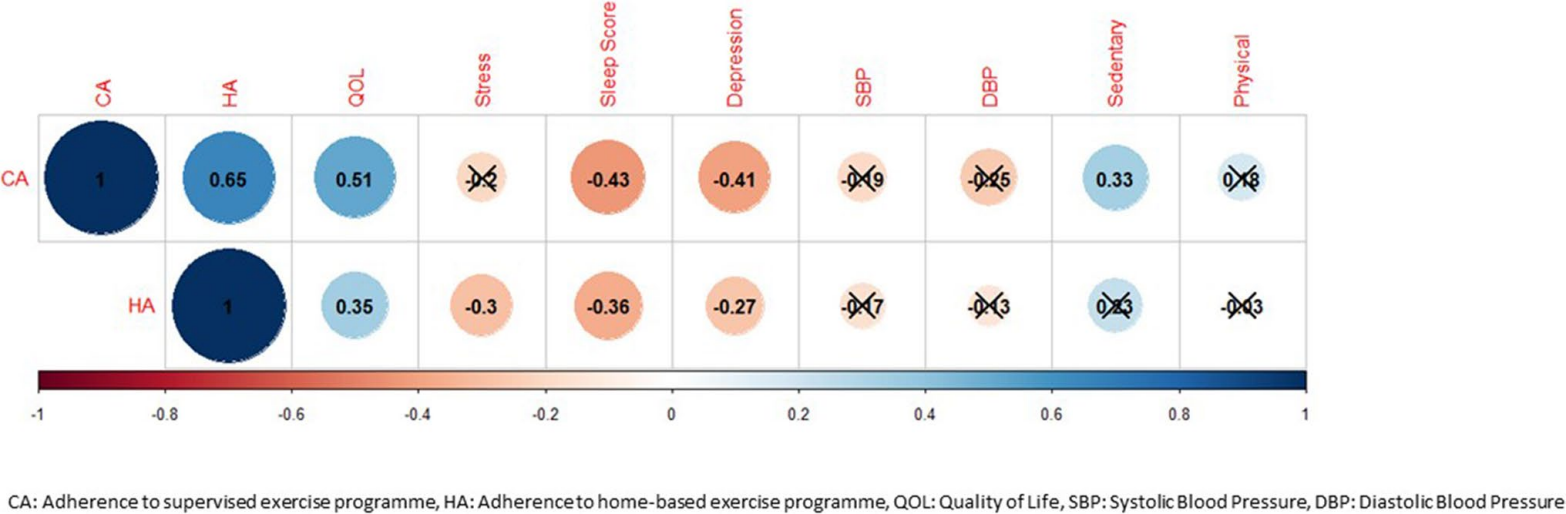
Combined correlation analysis for supervised and home-based exercise programmes at three timepoints

A total of 7 (21%) patients permanently discontinued aerobic training before week 24. Overall adherence to the supervised sessions was 83% (299 out of 360 possible sessions attended by participants). Patients attended on average 9.41 (SD 2.21) out of 12 supervised exercise sessions. Pain, shortness of breath, and conflicting medical appointments were the most common reasons given for missed exercise sessions. Participants were adherent to both the intensity (82%) and duration (83%) of the prescribed exercise programme during class sessions. A total of 21 (1%) supervised sessions, involving 9 (27%) patients, required early termination because of health-related non serious adverse events (e.g. excessive fatigue) or non-health-related reasons (e.g. difficulties with travel). No adverse events were reported by participants enrolled in this study. The combined correlation analysis for all three timepoints showed that adherence to supervised sessions was significantly correlated with quality of life, sleep score, depression, and sedentary behaviour; with the correlation being positive for quality of life and sedentary behaviour and negative for the other responses (Fig. 2).

Overall adherence to the non-supervised home exercise sessions (months 1–3), measured by heart rate monitors, was 72%. Participants were equally adherent to both the intensity (74%) and duration (71%) aspects of the prescribed home exercise programme. During the three unsupervised months of the programme (months 3–6), overall adherence to the home exercise programme was 67% (exercise intensity, 69%; duration, 65%). Exercise adherence levels in the intervention group did not correlate with patient-reported outcomes at month 3 or month 6. The combined correlation analysis for all three timepoints showed that adherence to non-supervised sessions was significantly correlated with quality of life, stress, sleep score, and depression; with the correlation being positive for quality of life and negative for stress, sleep score, and depression (Fig. 2).

### Intervention effects on quality of life

Modelling results for overall QOL scores showed that there was no significant main effect for the group (*p*-value = 0.6612) as well as for time (*p*-value = 0.6314) (Table 3). The *F*-test for the interaction effect too was insignificant (*p*-value = 0.5647). The Cohen’s d for the effect size (*d* =  − 0.082) also showed no effect of exercise on quality of life.

There was no significant main effect of the group on sleep score (*p*-value = 0.8653), stress (*p*-value = 0.3781), and depression (*p*-value = 0.2579). Improvements compared to the baseline (T0) were found for two responses at T3 (*sleep score*: coefficient =  − 0.82 (lower score = improved sleep), *p*-value = 0.0238; *depression*: coefficient =  − 1.25 (lower score = reduced depression), *p*-value = 0.0114) but there was no significant difference between the baseline and T6 for these responses (*sleep score*: *p*-value = 0.6813; *depression*: *p*-value = 0.0864). The interaction effect (group × time) was not significant for any of the three responses (*p*-values = 0.3286, 0.0724, and 0.6822 for sleep score, stress, and depression, respectively) (Table 4 and Supplemental Material). The Cohen’s d for the effect size suggested a negligible effect of exercise for sleep score (*d* =  − 0.25), stress (*d* = 0.2), and depression (*d* =  − 0.29) (Supplemental Material).

### Intervention effects on activity levels and cardiovascular health

Modelling results for overall physical activity scores showed that there was no significant main effect for the group (*p*-value = 0.25) or for time (*p*-value = 0.2422) (Supplemental Material). Modelling results for overall sedentary activity scores showed that there was a significant main effect for the group (coefficient = 503.89, *p*-value = 0.012) but not for time (*p*-value = 0.313) (Supplemental Material). The Cohen’s d for the effect size for physical activity (*d* =  − 0.284) and sedentary behaviour (*d* =  − 0.081) showed a negligible effect of the intervention on activity levels.

At baseline, 32 of the 67 (54%) participants were meeting the current ACSM exercise guidelines for patients living with cancer. The percentage of participants in the exercise group meeting exercise guidelines increased from 58% at months 0, to 66% at 6 months. The percentage of participants in the control group meeting the physical activity guidelines did not change over time (48% at baseline, 50% at month 3 and 48% at month 6).

Measures of systolic and diastolic blood pressure showed no significant difference between groups at baseline (*p* = 0.37 and *p* = 0.81, respectively). The Cohen’s d for the effect size for systolic blood pressure (*d* = 0.334) and diastolic blood pressure (*d* = 0.275) showed a negligible effect of the intervention on blood pressure levels (Supplemental Material). The Cohen’s d for the effect size for body mass index (*d* = 0.333) also showed a negligible effect of the intervention (Supplemental Material).

## Discussion

This study demonstrated that a 6-month aerobic exercise intervention was safe for a group of patients with a high burden of metastatic prostate cancer. While the exercise intervention did not show an effect amongst men with an already active lifestyle, the trial adds to the body of evidence examining the role of exercise for people with advanced disease.

Treatment and disease-related side effects as well as fear of skeletal fracture are likely to reduce physical activity levels in patients with bone metastatic prostate cancer [19]. Due to concerns of fragility fracture, exercise is often a perceived contraindication for patients with prostate cancer who present with bone metastases [20, 21]. However, this trial found that patients living with metastatic disease reported higher levels of self-reported physical activity levels (58% of participants in intervention group self-reported reaching 150 min of activity per week) than previous studies of men with metastatic prostate cancer ( approximately 29%) [22, 31]. However, physical activity levels in studies to date have been assessed using self-report measures, which may be affected by response and recall bias leading to both under and over-reporting [23]. Additionally, the relative wellness indicated in baseline patient reported outcomes was higher than in previous studies of patients with metastatic prostate cancer [22], suggesting data may not be representative of all bone metastatic prostate cancer patients. The ExPeCT trial did not exclude patients based on baseline physical activity levels. The lack of effect of the intervention on changing physical activity levels may be explained by participants’ high levels of baseline physical activity or the use of a subjective measure of physical activity. It may be those who experience the greatest morbidity arising from cancer or cancer treatment who benefit the most from physical activity interventions [24]. Future exercise trials that specifically include patients who are sedentary at baseline and use objective measurements of physical activity are needed to ensure that the results of trials can be appropriately applied to advanced cancer populations found in the clinical setting.

The absence of changes in quality-of-life outcomes in ExPeCT may be due to a number of factors. In all domains of quality of life (emotional, physical, function, and social/family well-being), the patient population in the ExPeCT trial reported higher mean quality-of-life scores than those found in normative data of male patients living with cancer [25]. It is possible that a ceiling effect was reached with these patients, possibly due to a prolonged treatment regime and ongoing medical follow-up; this has been reported in previous studies involving patients living with cancer [26, 27]. Many uncontrollable factors influence quality of life during advanced cancer, and a global measure of cancer-specific quality of life may be too broad to detect the likely narrower effects of exercise training [28]. Additionally, the literature regarding the effect of exercise on quality of life in patients with advanced cancer is inconsistent. While improvements in quality-of-life scores have been reported (29), the majority of papers report no change in outcomes [30, 31]. Alternative outcome measures, which consider the additional symptom burden associated with advanced cancer, may be more appropriate to capture change in health-related quality of life [32]. Future trials in advanced cancer populations should give careful consideration to the choice of quality-of-life outcome and instruments used to measure such outcomes.

Consistent with the results of other studies involving patients with advanced cancer, the ExPeCT exercise programme was well-tolerated by patients with metastatic bone disease, demonstrated by high adherence and low attrition rates [28, 33, 34]. The absence of any adverse events related to the exercise intervention in this study indicates that individualised physical activity programmes can be safely introduced for patients with many symptoms of advanced disease, including bone metastases. The exercise adherence rate reported in ExPeCT is higher than the values reported in exercise interventions involving patients receiving chemotherapy and is also within the common range reported by exercise trials involving older adults without cancer [35]. The findings support the current evidence that a combination of supervised exercise training with a requirement of independent self-directed exercise is likely to promote good adherence [36]. Additionally, the level of adherence to the exercise programme was maintained in the 3-month unsupervised exercise period, demonstrating that patients, when started on the programme, were able to continue exercising at home with remote monitoring.

There were no changes over time in the anthropometric variables measured in the ExPeCT study. The impact of exercise on measures of body composition in men with prostate cancer is inconsistent [28, 38]. The quantification of changes in body composition using BMI and girth measurements is difficult, and more precise measures, such as dual energy X-ray absorptiometry (DEXA) or MRI, are preferable to assess changes [34]. Indeed, a 12-week combined resistance and aerobic exercise intervention in non-metastatic patients with prostate cancer, using whole body and regional lean mass as primary endpoints, resulted in improvements in skeletal muscle mass via DEXA scanning [37]. The inclusion of resistance exercise may be an essential component of exercise interventions for this group to reverse the loss of muscle mass experienced by patients on androgen deprivation therapy [39]. Further examination of the efficacy of lifestyle interventions for evoking changes in body composition is important, as higher levels of body fat have been associated with higher grade tumours and disease progression [40]. Therefore, future studies should assess these parameters in metastatic populations by using precise anthropometric measurement techniques and incorporate a strengthening component in exercise interventions.

### Strengths and limitations

The current study has several strengths and limitations worthy of comment. It is one of the largest RCT’s evaluating the effects of exercise in patients with bone metastatic disease. The approach to exercise prescription in this study was patient inclusive, such that all patients can be prescribed some amount of exercise despite the presence of metastases. This method has significant potential for use in the clinical setting and adds to the recent paradigm shift in relation to exercise prescription in advanced prostate cancer [41]. A further strength of the current study is the objective measurement of adherence to the physical activity intervention in this metastatic population.

There are limitations to this study which warrant discussion. Current evidence suggests that resistance training is associated with clinically important positive effects on muscular function and body composition in patients during treatment or in long-term follow-up [42]. The aerobic intervention in the ExPeCT trial was not prescribed to target gains in these measures; however, the inclusion of resistance training may have resulted in improved outcomes post-intervention. Cancer progression this may have contributed to the missing effect, as the mean time from diagnosis was greater in the exercise arm than the control arm. Study inclusion criteria did not distinguish metastatic subtype; therefore, no subgroup analyses could be performed based on disease progression or treatment response. Finally, participants with high baseline levels of physical activity were not excluded from this study, which may have resulted in a sample not representative of the general advanced prostate cancer population.

## Conclusion

This study supports the safety and feasibility of exercise interventions in metastatic populations. However, contrary to the study hypotheses, an aerobic exercise intervention did not show an effect on cancer-specific quality of life amongst a group of physically active men with metastatic prostate cancer. Further work is needed to investigate the benefits associated with specific exercise modalities and how to optimise the prescription of exercise for patients living with advanced prostate cancer.

**Table 1:** Demographic characteristics at baseline. Results presented as mean ± s.d. or number of participants (percentage of participants)*. s.d*, *standard deviation*; *p value from χ2 test,* other p values from t test*

		Study arm		
Characteristic	Total study cohort (*n* = 61)	Exercise arm (*n* = 30)	Control arm (*n* = 31)	*p* value
Age (years)	69.4 ± 7.3	69.8 ± 7.0	69.9 ± 7.5	0.97
BMI (kg/m^2^)	29.2 ± 4.6	28.4 ± 4.84	29.9 ± 4.35	0.59
Waist circumference (cm)	102 ± 35.2	100.53 ± 14.62	104.13 ± 11.74	0.21
Systolic blood pressure (mm Hg)	139.35 (23.34)	141.07 (16.57)	136.17 (13.24)	0.37
Diastolic blood pressure (mm Hg)	78.67 (9.91)	78.37 (8.52)	78.70 (11.47)	0.81
Time since cancer diagnosis (months)	33.67 (32.61)	37.36 (32.30)	30.23 (33.07)	0.41
Current smoker, *n* (%)	5 (8)	5 (17)	0 (0)	0.01
Marital status, *n* (%)				
Married	37 (61)	15 (25)	22 (36)	0.13*
Widowed	11 (18)	8 (13)	3 (5)	
Divorced/separated	9 (15)	6 (10)	3 (5)	
Never married/not answered	4 (7)	1 (2)	3 (5)	
Work status *n* (%)				
Currently employed	7 (11)	2 (3)	5 (8)	0.18*
Retired	49 (80)	24 (39)	25 (41)	
Disability/unemployed	4 (7)	4 (7)	0 (0)	
Living arrangement, *n* (%)				
Alone	13 (21)	8 (13)	5 (8)	0.36*
With partner	39 (64)	16 (26)	23 (38)	
With other family	7 (11)	5 (8)	2 (3)	
Other	2 (3)	1 (2)	1 (2)	
Ethnicity, *n* (%)				
White/Caucasian	56 (92)	27 (44)	30 (49)	0.82*
Black/Afro-Carribean	3 (5)	2 (3)	1 (2)	
Asian	2 (3)	1 (2)	1 (2)

**Table 2:** Medical characteristics at baseline. s.d., standard deviation; MET, metabolic equivalent. *p value from *χ*2 test, other *p* values from *t* test

		Study arm		
Characteristic	Total study cohort (*n* = 61)	Exercise arm (*n* = 30)	Control arm (*n* = 31)	*p* value
Comorbidity, *n* (%)				
Hypertension	32 (52)	17 (28)	15 (25)	0.517
Hypercholesterolemia	25 (41)	12 (20)	13 (21)	0.684
Diabetes	15 (25)	7 (11)	8 (13)	0.766
CV disease	13 (21)	8 (13)	5 (8)	0.176
Severity of bone metastatic disease, *n* (%)				
Minor (1 region affected)	27 (44)	12 (20)	15 (25)	0.692*
Moderate (2 regions affected)	11 (18)	6 (10)	5 (8)	
Major (> 2 regions affected)	23 (38)	10 (16)	13(21)	
Gleason score, *n* (%)				
7	7 (11)	3 (5)	4 (6)	0.934*
8	20 (33)	9 (15)	11(18)	
9	26 (43)	15 (25)	11(18)	
Unknown	8 (13)	5 (8)	3 (5)	
Primary treatment, *n* (%)				
Hormones only	41 (67)	22 (36)	19 (31)	0.246
Radiation only	6 (10)	0	6 (10)	0.011
Hormones + radiation	8 (13)	5 (8)	3 (5)	0.412
Unknown	6 (10)	3 (5)	3 (5)	-
Achieving aerobic physical activity guidelines, *n* (%)				
Yes	32 (54)	17 (28)	15 (25)	0.73
No	29 (47)	13 (21)	16 (26)	
Overall physical activity level (MET-h/week)	36.95 ± 53.94	36.26 ± 42.70	37.63 ± 63.41	0.824
Overall daily sedentary activity levels (mins)	273.70 ± 260.85	270.74 ± 248.4	276.38 ± 275.29	0.347

**Table 3:** The effect of the exercise intervention on quality of life

Quality of life								
Main effects only					Raw data			
	Value	Standard error	*t*-value	*p*-value	Group	Time	Mean	SD
Intercept	121.4188	3.495383	34.73689	0	Control	0	119.96	20.733
Exercise	− 2.06983	4.714333	− 0.43905	0.6612	Control	3	122.06	21.112
Time_3	0.10983	1.815932	0.06048	0.9518	Control	6	125.12	21.525
Time_6	1.80667	2.412744	0.7488	0.455	Exercise	0	120.3	21.096
					Exercise	3	120.17	18.06
					Exercise	6	120.89	24.674

*Control group and 0 months are base levels for treatment and time respectively

**Table 4:** The effect of the exercise intervention on depression

Depression								
Main effects only					Raw data			
	Value	Standard error	*t*-value	*p*-value	Group	Time	Mean	SD
Intercept	3.224919	0.7201783	4.477945	0	Control	0	2.97	4.086
Exercise	0.942665	0.8305272	1.135019	0.2579	Control	3	2.04	2.261
Time_3	− 1.2459	0.4870307	− 2.55816	0.0114	Control	6	2.15	3.055
Time_6	− 0.78689	0.4564222	− 1.72403	0.0864	Exercise	0	4.43	5.171
					Exercise	3	3.04	4.614
					Exercise	6	3.68	5.065

*Control group and 0 months are base levels for treatment and time respectively

## Supplementary information

Below is the link to the electronic supplementary material.

Supplementary file1 (DOCX 93 KB)

## Data Availability

The data that support the findings of this study are available from the corresponding author, GS, upon reasonable request.

## Code availability

N/a.
