# Supplementary material for: ExPeCT: a randomised trial examining the impact of exercise on quality of life in men with metastatic prostate cancer
Source: Support Care Cancer. 2023 Apr 22;31(5):292. doi: 10.1007/s00520-023-07740-4 (PMC10122616; doi:10.1007/s00520-023-07740-4)

|  |  |  |  |  |  |  |  |  |  |  |  |  |  |  |  |  |  |  |  |  |  |  |  |  |  |  |  |  |  |
| --- | --- | --- | --- | --- | --- | --- | --- | --- | --- | --- | --- | --- | --- | --- | --- | --- | --- | --- | --- | --- | --- | --- | --- | --- | --- | --- | --- | --- | --- |
|  | **Sleep Score** | |  |  |  |  |  |  |  |  |  |  |  |  |  |  |  |  |  |  |  |  |  |  |  |  |  |  |  |
|  |  |  |  |  |  |  |  |  |  |  |  |  |  |  |  |  |  |  |  |  |  |  |  |  |  |  |  |  |  |
|  | **Main Effects Only** |  |  |  |  |  |  |  |  |  |  |  |  |  |  |  |  |  |  |  |  |  |  |  |  |  |  |  |  |
|  |  | **Value** | **Standard Error** | **t-value** | **p-value** |  |  |  |  |  |  |  |  |  |  |  |  |  |  |  |  |  |  |  |  |  |  |  |  |
|  | **Intercept** | 6.977717 | 0.6715614 | 10.39029 | 0 |  |  |  | **Raw Data** |  |  |  |  |  |  |  |  |  |  |  |  |  |  |  |  |  |  |  |  |
|  | **Exercise** | -0.15469 | 0.9109395 | -0.16982 | 0.8653 |  |  |  | **Group** | **Time** | **Mean** | **SD** |  |  |  |  |  |  |  |  |  |  |  |  |  |  |  |  |  |
|  | **Time_3** | -0.81787 | 0.3587104 | -2.28003 | 0.0238 |  |  |  | Control | 0 | 6.77 | 3.93 |  |  |  |  |  |  |  |  |  |  |  |  |  |  |  |  |  |
|  | **Time_6** | -0.14754 | 0.3587104 | -0.41131 | 0.6813 |  |  |  | Control | 3 | 6.41 | 3.64 |  |  |  |  |  |  |  |  |  |  |  |  |  |  |  |  |  |
|  | *Control group and 0 months are base levels for treatment and time respectively | | | | | |  |  | Control | 6 | 6.87 | 3.709 |  |  |  |  |  |  |  |  |  |  |  |  |  |  |  |  |  |
|  |  |  |  |  |  |  |  |  | Exercise | 0 | 7.03 | 3.899 |  |  |  |  |  |  |  |  |  |  |  |  |  |  |  |  |  |
|  | **Test for Interaction Effect** |  | |  |  |  |  |  | Exercise | 3 | 5.96 | 4.372 |  |  |  |  |  |  |  |  |  |  |  |  |  |  |  |  |  |
|  |  | **df** | **LogLik** | **L Ratio** | **p-value** |  |  |  | Exercise | 6 | 6.86 | 5.023 |  |  |  |  |  |  |  |  |  |  |  |  |  |  |  |  |  |
|  | **Main Effects Model** | 6 | -451.96 |  |  |  |  |  |  |  |  |  |  |  |  |  |  |  |  |  |  |  |  |  |  |  |  |  |  |
|  | **Interaction Effects** | 8 | -450.84 | 2.226 | 0.3286 |  |  |  |  |  |  |  |  |  |  |  |  |  |  |  |  |  |  |  |  |  |  |  |  |
|  |  |  |  |  |  |  |  |  |  |  |  |  |  |  |  |  |  |  |  |  |  |  |  |  |  |  |  |  |  |
|  |  |  |  | **Cohen's Distance for effect of treatment on Sleep Score = -0.2521453** | | | | | | |  |  |  |  |  |  |  |  |  |  |  |  |  |  |  |  |  |  |  |
|  |  |  |  |  |  |  |  |  |  |  |  |  |  |  |  |  |  |  |  |  |  |  |  |  |  |  |  |  |  |
|  |  |  |  |  |  |  |  |  |  |  |  |  |  |  |  |  |  |  |  |  |  |  |  |  |  |  |  |  |  |
|  | **Stress** |  |  |  |  |  |  |  |  |  |  |  |  |  |  |  |  |  |  |  |  |  |  |  |  |  |  |  |  |
|  |  |  |  |  |  |  |  |  |  |  |  |  |  |  |  |  |  |  |  |  |  |  |  |  |  |  |  |  |  |
|  | **Main Effects Only** |  |  |  |  |  |  |  |  |  |  |  |  |  |  |  |  |  |  |  |  |  |  |  |  |  |  |  |  |
|  |  | **Value** | **Standard Error** | **t-value** | **p-value** |  |  |  |  |  |  |  |  |  |  |  |  |  |  |  |  |  |  |  |  |  |  |  |  |
|  | **Intercept** | 3.59767 | 0.4926378 | 7.302871 | 0 |  |  |  |  |  |  |  |  |  |  |  |  |  |  |  |  |  |  |  |  |  |  |  |  |
|  | **Exercise** | -0.58139 | 0.6580084 | -0.88356 | 0.3781 |  |  |  | **Raw Data** |  |  |  |  |  |  |  |  |  |  |  |  |  |  |  |  |  |  |  |  |
|  | **Time_3** | 0.8 | 0.3166417 | 2.526515 | 0.0124 |  |  |  | **Group** | **Time** | **Mean** | **SD** |  |  |  |  |  |  |  |  |  |  |  |  |  |  |  |  |  |
|  | **Time_6** | 0.116667 | 0.3166417 | 0.36845 | 0.713 |  |  |  | Control | 0 | 3.74 | 2.816 |  |  |  |  |  |  |  |  |  |  |  |  |  |  |  |  |  |
|  | *Control group and 0 months are base levels for treatment and time respectively | | | | | |  |  | Control | 3 | 4.54 | 3.114 |  |  |  |  |  |  |  |  |  |  |  |  |  |  |  |  |  |
|  |  |  |  |  |  |  |  |  | Control | 6 | 3.08 | 2.812 |  |  |  |  |  |  |  |  |  |  |  |  |  |  |  |  |  |
|  | **Test for Interaction Effect** |  | |  |  |  |  |  | Exercise | 0 | 2.86 | 3.43 |  |  |  |  |  |  |  |  |  |  |  |  |  |  |  |  |  |
|  |  | **df** | **LogLik** | **L Ratio** | **p-value** |  |  |  | Exercise | 3 | 3.48 | 2.874 |  |  |  |  |  |  |  |  |  |  |  |  |  |  |  |  |  |
|  | **Main Effects Model** | 6 | -408.51 |  |  |  |  |  | Exercise | 6 | 3.45 | 3.128 |  |  |  |  |  |  |  |  |  |  |  |  |  |  |  |  |  |
|  | **Interaction Effects** | 8 | -405.89 | 5.2498 | 0.0724 |  |  |  |  |  |  |  |  |  |  |  |  |  |  |  |  |  |  |  |  |  |  |  |  |
|  |  |  |  |  |  |  |  |  |  |  |  |  |  |  |  |  |  |  |  |  |  |  |  |  |  |  |  |  |  |
|  |  |  |  | **Cohen's Distance for effect of treatment on Stress = 0.1991432** | | | | | | |  |  |  |  |  |  |  |  |  |  |  |  |  |  |  |  |  |  |  |
|  |  |  |  |  |  |  |  |  |  |  |  |  |  |  |  |  |  |  |  |  |  |  |  |  |  |  |  |  |  |
|  |  |  |  |  |  |  |  |  |  |  |  |  |  |  |  |  |  |  |  |  |  |  |  |  |  |  |  |  |  |
|  | **Systolic Blood Pressure** | | |  |  |  |  |  |  |  |  |  |  |  |  |  |  |  |  |  |  |  |  |  |  |  |  |  |  |
|  |  |  |  |  |  |  |  |  |  |  |  |  |  |  |  |  |  |  |  |  |  |  |  |  |  |  |  |  |  |
|  | **Main Effects Only** |  |  |  |  |  |  |  |  |  |  |  |  |  |  |  |  | **Interaction Effect** | |  |  |  |  |  |  |  |  |  |  |
|  |  | **Value** | **Standard Error** | **t-value** | **p-value** |  |  |  |  |  |  |  |  |  |  |  |  |  | **Value** | **Standard Error** | **t-value** | **p-value** |  |  |  |  |  |  |  |
|  | **Intercept** | 140.3668 | 2.52267 | 55.64216 | 0 |  |  |  | **Raw Data** |  |  |  |  |  |  |  |  | **Intercept** | 136.1667 | 2.815808 | 48.35793 | 0 |  |  |  |  |  |  |  |
|  | **Exercise** | -3.50026 | 2.928337 | -1.19531 | 0.2336 |  |  |  | **Group** | **Time** | **Mean** | **SD** |  |  |  |  |  | **Exercise** | 4.9 | 3.982154 | 1.23049 | 0.2202 |  |  |  |  |  |  |  |
|  | **Time_3** | -1.7 | 2.512386 | -0.67665 | 0.4995 |  |  |  | Control | 0 | 136.17 | 14.184 |  |  |  |  |  | **Time_3** | 5.53333 | 3.311361 | 1.67101 | 0.0965 |  |  |  |  |  |  |  |
|  | **Time_6** | -0.21667 | 2.324276 | -0.09322 | 0.9258 |  |  |  | Control | 3 | 141.43 | 15.782 |  |  |  |  |  | **Time_6** | 7.43333 | 2.9851 | 2.49015 | 0.0137 |  |  |  |  |  |  |  |
|  | *Control group and 0 months are base levels for treatment and time respectively | | | | | |  |  | Control | 6 | 143.39 | 18.51 |  |  |  |  |  | **Exercise*Time_3** | -14.4667 | 4.682972 | -3.08921 | 0.0023 |  |  |  |  |  |  |  |
|  |  |  |  |  |  |  |  |  | Exercise | 0 | 141.07 | 16.569 |  |  |  |  |  | **Exercise*Time_6** | -15.3 | 4.221569 | -3.62424 | 0.0004 |  |  |  |  |  |  |  |
|  | **Test for Interaction Effect** |  | |  |  |  |  |  | Exercise | 3 | 129.79 | 12.864 |  |  |  |  |  |  |  |  |  |  |  |  |  |  |  |  |  |
|  |  | **df** | **LogLik** | **L Ratio** | **p-value** |  |  |  | Exercise | 6 | 131.14 | 13.778 |  |  |  |  |  |  |  |  |  |  |  |  |  |  |  |  |  |
|  | **Main Effects Model** | 10 | -712.39 |  |  |  |  |  |  |  |  |  |  |  |  |  |  |  |  |  |  |  |  |  |  |  |  |  |  |
|  | **Interaction Effects** | 12 | -706.25 | 12.281 | 0.0022 |  |  |  |  |  |  |  |  |  |  |  |  |  |  |  |  |  |  |  |  |  |  |  |  |
|  |  |  |  |  |  |  |  |  |  |  |  |  |  |  |  |  |  | *Control group and 0 months are base levels for treatment and time respectively | | | | | | | |  |  |  |  |
|  |  |  | **Cohen's Distance for effect of treatment on Systolic Blood Pressure = 0.3349198** | | | | | | | | |  |  |  |  |  |  |  |  |  |  |  |  |  |  |  |  |  |  |
|  |  |  |  |  |  |  |  |  |  |  |  |  |  |  |  |  |  |  |  |  |  |  |  |  |  |  |  |  |  |
|  |  |  |  |  |  |  |  |  |  |  |  |  |  |  |  |  |  |  |  |  |  |  |  |  |  |  |  |  |  |
|  | **Diastolic Blood Pressure** | | |  |  |  |  |  |  |  |  |  |  |  |  |  |  |  |  |  |  |  |  |  |  |  |  |  |  |
|  |  |  |  |  |  |  |  |  |  |  |  |  |  |  |  |  |  |  |  |  |  |  |  |  |  |  |  |  |  |
|  | **Main Effects Only** |  |  |  |  |  |  |  |  |  |  |  |  |  |  |  |  |  |  |  |  |  |  |  |  |  |  |  |  |
|  |  | **Value** | **Standard Error** | **t-value** | **p-value** |  |  |  |  |  |  |  |  |  |  |  |  |  |  |  |  |  |  |  |  |  |  |  |  |
|  | **Intercept** | 79.7975 | 1.532848 | 52.05834 | 0 |  |  |  | **Raw Data** |  |  |  |  |  |  |  |  |  |  |  |  |  |  |  |  |  |  |  |  |
|  | **Exercise** | -2.52834 | 1.608618 | -1.57174 | 0.1178 |  |  |  | **Group** | **Time** | **Mean** | **SD** |  |  |  |  |  |  |  |  |  |  |  |  |  |  |  |  |  |
|  | **Time_3** | -0.15 | 1.464508 | -0.10242 | 0.9185 |  |  |  | Control | 0 | 78.7 | 11.469 |  |  |  |  |  |  |  |  |  |  |  |  |  |  |  |  |  |
|  | **Time_6** | -0.08333 | 1.368745 | -0.06088 | 0.9515 |  |  |  | Control | 3 | 79.04 | 9.113 |  |  |  |  |  |  |  |  |  |  |  |  |  |  |  |  |  |
|  | *Control group and 0 months are base levels for treatment and time respectively | | | | | |  |  | Control | 6 | 79.48 | 7.669 |  |  |  |  |  |  |  |  |  |  |  |  |  |  |  |  |  |
|  |  |  |  |  |  |  |  |  | Exercise | 0 | 78.37 | 8.524 |  |  |  |  |  |  |  |  |  |  |  |  |  |  |  |  |  |
|  | **Test for Interaction Effect** |  | |  |  |  |  |  | Exercise | 3 | 76.62 | 6.768 |  |  |  |  |  |  |  |  |  |  |  |  |  |  |  |  |  |
|  |  | **df** | **LogLik** | **L Ratio** | **p-value** |  |  |  | Exercise | 6 | 76.1 | 7.648 |  |  |  |  |  |  |  |  |  |  |  |  |  |  |  |  |  |
|  | **Main Effects Model** | 10 | -594.55 |  |  |  |  |  |  |  |  |  |  |  |  |  |  |  |  |  |  |  |  |  |  |  |  |  |  |
|  | **Interaction Effects** | 12 | -593.9 | 1.3062 | 0.5204 |  |  |  |  |  |  |  |  |  |  |  |  |  |  |  |  |  |  |  |  |  |  |  |  |
|  |  |  |  |  |  |  |  |  |  |  |  |  |  |  |  |  |  |  |  |  |  |  |  |  |  |  |  |  |  |
|  |  |  | **Cohen's Distance for effect of treatment on Diastolic Blood Pressure = 0.2751302** | | | | | | | | | |  |  |  |  |  |  |  |  |  |  |  |  |  |  |  |  |  |
|  |  |  |  |  |  |  |  |  |  |  |  |  |  |  |  |  |  |  |  |  |  |  |  |  |  |  |  |  |  |
|  |  |  |  |  |  |  |  |  |  |  |  |  |  |  |  |  |  |  |  |  |  |  |  |  |  |  |  |  |  |
|  | **Body Mass Index** | |  |  |  |  |  |  |  |  |  |  |  |  |  |  |  |  |  |  |  |  |  |  |  |  |  |  |  |
|  |  |  |  |  |  |  |  |  |  |  |  |  |  |  |  |  |  |  |  |  |  |  |  |  |  |  |  |  |  |
|  | **Main Effects Only** |  |  |  |  |  |  |  |  |  |  |  |  |  |  |  |  |  |  |  |  |  |  |  |  |  |  |  |  |
|  |  | **Value** | **Standard Error** | **t-value** | **p-value** |  |  |  |  |  |  |  |  |  |  |  |  |  |  |  |  |  |  |  |  |  |  |  |  |
|  | **Intercept** | 29.94084 | 0.8138486 | 36.78921 | 0 |  |  |  | **Raw Data** |  |  |  |  |  |  |  |  |  |  |  |  |  |  |  |  |  |  |  |  |
|  | **Exercise** | -1.49972 | 1.1553019 | -1.29812 | 0.1959 |  |  |  | **Group** | **Time** | **Mean** | **SD** |  |  |  |  |  |  |  |  |  |  |  |  |  |  |  |  |  |
|  | **Time_3** | -0.03771 | 0.1093698 | -0.34475 | 0.7307 |  |  |  | Control | 0 | 29.93 | 4.3 |  |  |  |  |  |  |  |  |  |  |  |  |  |  |  |  |  |
|  | **Time_6** | 0.167213 | 0.1539896 | 1.08587 | 0.279 |  |  |  | Control | 3 | 29.65 | 4.364 |  |  |  |  |  |  |  |  |  |  |  |  |  |  |  |  |  |
|  | *Control group and 0 months are base levels for treatment and time respectively | | | | | |  |  | Control | 6 | 29.99 | 4.602 |  |  |  |  |  |  |  |  |  |  |  |  |  |  |  |  |  |
|  |  |  |  |  |  |  |  |  | Exercise | 0 | 28.45 | 4.846 |  |  |  |  |  |  |  |  |  |  |  |  |  |  |  |  |  |
|  | **Test for Interaction Effect** |  | |  |  |  |  |  | Exercise | 3 | 28.47 | 4.93 |  |  |  |  |  |  |  |  |  |  |  |  |  |  |  |  |  |
|  |  | **df** | **LogLik** | **L Ratio** | **p-value** |  |  |  | Exercise | 6 | 28.69 | 4.741 |  |  |  |  |  |  |  |  |  |  |  |  |  |  |  |  |  |
|  | **Main Effects Model** | 6 | -330.31 |  |  |  |  |  |  |  |  |  |  |  |  |  |  |  |  |  |  |  |  |  |  |  |  |  |  |
|  | **Interaction Effects** | 8 | -330.23 | 0.175 | 0.9162 |  |  |  |  |  |  |  |  |  |  |  |  |  |  |  |  |  |  |  |  |  |  |  |  |
|  |  |  |  |  |  |  |  |  |  |  |  |  |  |  |  |  |  |  |  |  |  |  |  |  |  |  |  |  |  |
|  |  |  | **Cohen's Distance for effect of treatment on Body Mass Index = 0.333072** | | | | | | | | |  |  |  |  |  |  |  |  |  |  |  |  |  |  |  |  |  |  |
|  |  |  |  |  |  |  |  |  |  |  |  |  |  |  |  |  |  |  |  |  |  |  |  |  |  |  |  |  |  |
|  |  |  |  |  |  |  |  |  |  |  |  |  |  |  |  |  |  |  |  |  |  |  |  |  |  |  |  |  |  |
|  | **Physical Activity** | |  |  |  |  |  |  |  |  |  |  |  |  |  |  |  |  |  |  |  |  |  |  |  |  |  |  |  |
|  |  |  |  |  |  |  |  |  |  |  |  |  |  |  |  |  |  |  |  |  |  |  |  |  |  |  |  |  |  |
|  | **Main Effects Only** |  |  |  |  |  |  |  |  |  |  |  |  |  |  |  |  |  |  |  |  |  |  |  |  |  |  |  |  |
|  |  | **Value** | **Standard Error** | **t-value** | **p-value** |  |  |  |  |  |  |  |  |  |  |  |  |  |  |  |  |  |  |  |  |  |  |  |  |
|  | **Intercept** | 27.11104 | 8.110821 | 3.342577 | 0.001 |  |  |  | **Raw Data** |  |  |  |  |  |  |  |  |  |  |  |  |  |  |  |  |  |  |  |  |
|  | **Exercise** | 10.54164 | 9.185766 | 1.147606 | 0.2527 |  |  |  | **Group** | **Time** | **Mean** | **SD** |  |  |  |  |  |  |  |  |  |  |  |  |  |  |  |  |  |
|  | **Time_3** | 0.363 | 7.87333 | 0.046105 | 0.9633 |  |  |  | Control | 0 | 33.62 | 64.58 |  |  |  |  |  |  |  |  |  |  |  |  |  |  |  |  |  |
|  | **Time_6** | 11.90383 | 9.752083 | 1.220645 | 0.2239 |  |  |  | Control | 3 | 27.31 | 37.219 |  |  |  |  |  |  |  |  |  |  |  |  |  |  |  |  |  |
|  | *Control group and 0 months are base levels for treatment and time respectively | | | | | |  |  | Control | 6 | 31.65 | 37.859 |  |  |  |  |  |  |  |  |  |  |  |  |  |  |  |  |  |
|  |  |  |  |  |  |  |  |  | Exercise | 0 | 31.54 | 37.102 |  |  |  |  |  |  |  |  |  |  |  |  |  |  |  |  |  |
|  | **Test for Interaction Effect** |  | |  |  |  |  |  | Exercise | 3 | 41.22 | 53.028 |  |  |  |  |  |  |  |  |  |  |  |  |  |  |  |  |  |
|  |  | **df** | **LogLik** | **L Ratio** | **p-value** |  |  |  | Exercise | 6 | 65.1 | 106.017 |  |  |  |  |  |  |  |  |  |  |  |  |  |  |  |  |  |
|  | **Main Effects Model** | 10 | -952.82 |  |  |  |  |  |  |  |  |  |  |  |  |  |  |  |  |  |  |  |  |  |  |  |  |  |  |
|  | **Interaction Effects** | 12 | -951.39 | 2.87 | 0.2381 |  |  |  |  |  |  |  |  |  |  |  |  |  |  |  |  |  |  |  |  |  |  |  |  |
|  |  |  |  |  |  |  |  |  |  |  |  |  |  |  |  |  |  |  |  |  |  |  |  |  |  |  |  |  |  |
|  |  |  | **Cohen's Distance for effect of treatment on Physical Activity = -0.2841268** | | | | | | | | |  |  |  |  |  |  |  |  |  |  |  |  |  |  |  |  |  |  |
|  |  |  |  |  |  |  |  |  |  |  |  |  |  |  |  |  |  |  |  |  |  |  |  |  |  |  |  |  |  |
|  |  |  |  |  |  |  |  |  |  |  |  |  |  |  |  |  |  |  |  |  |  |  |  |  |  |  |  |  |  |
|  | **Sedentary Behaviour** | |  |  |  |  |  |  |  |  |  |  |  |  |  |  |  |  |  |  |  |  |  |  |  |  |  |  |  |
|  |  |  |  |  |  |  |  |  |  |  |  |  |  |  |  |  |  |  |  |  |  |  |  |  |  |  |  |  |  |
|  | **Main Effects Only** |  |  |  |  |  |  |  |  |  |  |  |  |  |  |  |  | **Interaction Effect** | |  |  |  |  |  |  |  |  |  |  |
|  |  | **Value** | **Standard Error** | **t-value** | **p-value** |  |  |  |  |  |  |  |  |  |  |  |  |  | **Value** | **Standard Error** | **t-value** | **p-value** |  |  |  |  |  |  |  |
|  | **Intercept** | 1385.899 | 248.0137 | 5.587995 | 0 |  |  |  | **Raw Data** |  |  |  |  |  |  |  |  | **Intercept** | 1623.048 | 316.8686 | 5.12215 | 0 |  |  |  |  |  |  |  |
|  | **Exercise** | 503.8979 | 198.4451 | 2.53923 | 0.012 |  |  |  | **Group** | **Time** | **Mean** | **SD** |  |  |  |  |  | **Exercise** | 13.2447 | 455.7805 | 0.029059 | 0.9769 |  |  |  |  |  |  |  |
|  | **Time_3** | -155.067 | 275.9117 | -0.56202 | 0.5748 |  |  |  | Control | 0 | 1623.05 | 1782.08 |  |  |  |  |  | **Time_3** | 122.2258 | 382.4806 | 0.319561 | 0.7497 |  |  |  |  |  |  |  |
|  | **Time_6** | -353.267 | 239.5874 | -1.47448 | 0.1421 |  |  |  | Control | 3 | 1834.25 | 1949.571 |  |  |  |  |  | **Time_6** | -783.145 | 325.2476 | -2.40784 | 0.0171 |  |  |  |  |  |  |  |
|  | *Control group and 0 months are base levels for treatment and time respectively | | | | | |  |  | Control | 6 | 919.79 | 748.532 |  |  |  |  |  | **Exercise*Time_3** | -573.709 | 550.1562 | -1.04281 | 0.2985 |  |  |  |  |  |  |  |
|  |  |  |  |  |  |  |  |  | Exercise | 0 | 1636.29 | 1744.938 |  |  |  |  |  | **Exercise*Time_6** | 889.4038 | 467.8329 | 1.901114 | 0.0589 |  |  |  |  |  |  |  |
|  | **Test for Interaction Effect** |  | |  |  |  |  |  | Exercise | 3 | 1216.93 | 1132.337 |  |  |  |  |  |  |  |  |  |  |  |  |  |  |  |  |  |
|  |  | **df** | **LogLik** | **L Ratio** | **p-value** |  |  |  | Exercise | 6 | 1882 | 1258.372 |  |  |  |  |  |  |  |  |  |  |  |  |  |  |  |  |  |
|  | **Main Effects Model** | 10 | -1550.91 |  |  |  |  |  |  |  |  |  |  |  |  |  |  |  |  |  |  |  |  |  |  |  |  |  |  |
|  | **Interaction Effects** | 12 | -1545.07 | 11.6744 | 0.0029 |  |  |  |  |  |  |  |  |  |  |  |  |  |  |  |  |  |  |  |  |  |  |  |  |
|  |  |  |  |  |  |  |  |  |  |  |  |  |  |  |  |  |  | *Control group and 0 months are base levels for treatment and time respectively | | | | | | | |  |  |  |  |
|  |  |  | **Cohen's Distance for effect of treatment on Sedentary Behaviour = -0.08140236** | | | | | | | | |  |  |  |  |  |  |  |  |  |  |  |  |  |  |  |  |  |  |
|  |  |  |  |  |  |  |  |  |  |  |  |  |  |  |  |  |  |  |  |  |  |  |  |  |  |  |  |  |  |

**Depression**


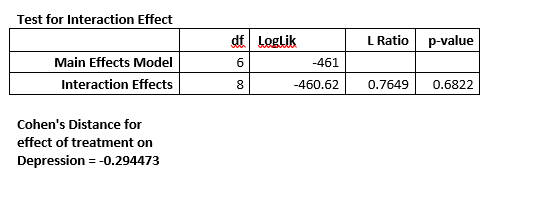


**Quality of Life**


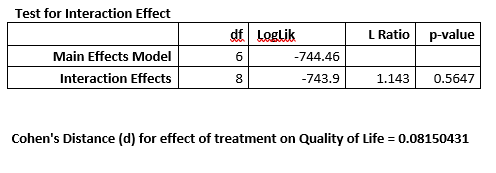

Supplement: Supplementary file 1 — Supplementary file1 (DOCX 93 KB) [file 520_2023_7740_MOESM1_ESM.docx]
